# Memantine Monotherapy for Alzheimer’s Disease: A Systematic Review and Meta-Analysis

**DOI:** 10.1371/journal.pone.0123289

**Published:** 2015-04-10

**Authors:** Shinji Matsunaga, Taro Kishi, Nakao Iwata

**Affiliations:** Department of Psychiatry, Fujita Health University School of Medicine, Toyoake, Aichi, Japan; University of Glasgow, UNITED KINGDOM

## Abstract

**Background:**

We performed an updated meta-analysis of randomized placebo-controlled trials testing memantine monotherapy for patients with Alzheimer’s disease (AD).

**Methods:**

The meta-analysis included randomized controlled trials of memantine monotherapy for AD, omitting those in which patients were also administered a cholinesterase inhibitor. Cognitive function, activities of daily living, behavioral disturbances, global function, stage of dementia, drug discontinuation rate, and individual side effects were compared between memantine monotherapy and placebo groups. The primary outcomes were cognitive function and behavioral disturbances; the others were secondary outcomes.

**Results:**

Nine studies including 2433 patients that met the study’s inclusion criteria were identified. Memantine monotherapy significantly improved cognitive function [standardized mean difference (SMD)=−0.27, 95% confidence interval (CI)=−0.39 to −0.14, p=0.0001], behavioral disturbances (SMD=−0.12, 95% CI=−0.22 to −0.01, p=0.03), activities of daily living (SMD=−0.09, 95% CI=−0.19 to −0.00, p=0.05), global function assessment (SMD=−0.18, 95% CI=−0.27 to −0.09, p=0.0001), and stage of dementia (SMD=−0.23, 95% CI=−0.33 to −0.12, p=0.0001) scores. Memantine was superior to placebo in terms of discontinuation because of inefficacy [risk ratio (RR)=0.36, 95% CI=0.17¬ to 0.74, p=0.006, number needed to harm (NNH)=non significant]. Moreover, memantine was associated with less agitation compared with placebo (RR=0.68, 95% CI=0.49 to 0.94, p=0.02, NNH=non significant). There were no significant differences in the rate of discontinuation because of all causes, all adverse events, and individual side effects other than agitation between the memantine monotherapy and placebo groups.

**Conclusions:**

Memantine monotherapy improved cognition, behavior, activities of daily living, global function, and stage of dementia and was well-tolerated by AD patients. However, the effect size in terms of efficacy outcomes was small and thus there is limited evidence of clinical benefit.

## Introduction

Dementia is not only a significant individual health problem but also a societal burden. Alzheimer’s Disease International reported that over 35 million individuals worldwide currently live with dementia (http://wwwalzcouk/research/world-report). Alzheimer’s disease (AD) is a neurodegenerative disorder characterized by progressive loss of cognitive function and other neurobehavioral symptoms. The pathology of AD includes the accumulation of extracellular senile plaques composed primarily of β-amyloid and intracellular neurofibrillary tangles comprising abnormally hyperphosphorylated tau, a microtubule-associated protein [1].

Currently, memantine is available in Japan for the treatment of AD and was approved for the treatment of moderate-to-severe AD by the United States Food and Drug Administration (FDA). Memantine is believed to exert its therapeutic effect by acting as a low-to-moderate affinity, non-competitive N-methyl-D-aspartate (NMDA) receptor antagonist that binds preferentially to open NMDA receptor-operated calcium channels [2, 3]. This activity-dependent binding blocks NMDA-mediated ion flux and ameliorates the deleterious effects of sustained, pathologically elevated levels of glutamate (excitotoxicity) that may lead to neuronal dysfunction [4]. The efficacy of memantine in the management of patients with AD, vascular dementia, and mixed dementia was assessed in a Cochrane meta-analysis including 12 randomized controlled trials (RCTs) [5]. The meta-analysis showed that memantine was superior to placebo in benefiting cognitive function for mild-to-moderate AD and moderate-to-severe AD [mild-to-moderate AD, using Alzheimer’s Disease Assessment Scale cognitive subscale (ADAS-cog) [6], weighted mean difference (WMD) = −0.99, 95% confidence interval (CI) = −0.21 to −1.78, p = 0.013, *I* ^2^ = 0%, 3 studies, n = 1279; moderate-to-severe AD, using Severe Impairment Battery (SIB) [7], WMD = −2.97, 95% CI = −1.68 to −4.26, p = 0.00001, *I* ^2^ = 74%, 3 studies, n = 976]. However, the meta-analysis included both memantine monotherapy studies and memantine—cholinesterase inhibitor (ChEI) combination therapy studies. To clarify the clinical pharmacological characteristic of memantine, a meta-analysis should be conducted using data only from memantine monotherapy studies in patients with AD. A recent meta-analysis of 2 studies including 572 patients [8] suggested that memantine monotherapy did not significantly benefit moderate-to-severe AD as assessed by the Neuropsychiatric Inventory (NPI) [9] (WMD = −1.65, 95% CI = −4.78 to 1.49, p = 0.247, *I* ^2^ = 25.3%, 2 studies, n = not reported). However, apart from the small number of studies and patients, this meta-analysis did not evaluate other outcome measures such as cognitive function, activities of daily living, and global function assessment scores. To clarify whether memantine monotherapy is more efficacious than placebo for patients with AD, we have performed a meta-analysis of memantine monotherapy for AD including 9 studies with a total of 2433 patients.

## Materials and Methods

This meta-analysis was performed according to the Preferred Reporting Items for Systematic Reviews and Meta-Analyses (PRISMA) guidelines [10] (S1 PRISMA Checklist). We performed a systematic literature review using the PICO strategy (Patients: AD, Intervention: memantine monotherapy, Comparator: placebo or usual care, and Outcome: cognitive function, activities of daily living, behavioral disturbances, global function, stage of dementia, drug discontinuation rate, and individual side effects).

### Inclusion Criteria, Search Strategy, Data Extraction, and Outcome Measures

We included RCTs of memantine monotherapy for patients with AD, omitting those in which patients were also administered a ChEI, and included studies that were not double blinded and/or placebo controlled (i.e., conventional treatment regimen) in order to obtain a larger patient population exposed to the drug. To identify relevant studies, we searched PubMed, the Cochrane Library databases, Google Scholar, EMBASE, CINAHL, and PsycINFO citations. There were no language restrictions, and we considered all studies published up to October 31, 2014. We used the following key words: “memantine” AND “Alzheimer’s disease” OR “Alzheimer disease.” Additional eligible studies were sought by searching the reference lists from primary articles and relevant reviews.

Two authors (S.M. and T.K.) of this meta-analysis scrutinized the patient inclusion and exclusion criteria for the identified studies. When data required for the meta-analysis were missing, the first and/or corresponding authors were contacted for additional information, including endpoint scores. All 3 authors of the current study independently extracted, checked, and entered the data into Review Manager (Version 5.2 for Windows, Cochrane Collaboration, http://ims.cochrane.org/revman). Discrepancies in different coding forms were resolved by discussions between authors (S.M. and T.K.)

### Data Synthesis and Statistical Analysis

Each outcome measure reported here was used in at least 3 out of the 9 included studies. The primary outcome measures for efficacy were cognitive function and behavioral disturbances associated with AD. Cognitive function was measured using the SIB, ADAS-cog, Standardized Mini-Mental State Examination (SMMSE) [11], or MMSE [12]. Two studies [13, 14] used 2 cognitive function scales (SIB and MMSE) and 1 study [15] used the SIB, ADAS-cog, and MMSE scales. Because SIB was the most common instrument used in the studies included in the current meta-analysis for evaluating cognitive function, we used data of SIB when multiple scales were used for this outcome (S1 Appendix). Behavioral disturbances were measured using the NPI and Behavioral Pathology in Alzheimer’s Disease Rating Scale (Behave-AD) scores [16]. Secondary outcome measures included activities of daily living [Alzheimer’s Disease Cooperative Study-Activities of Daily Living Inventory (modified for more severe dementia) (ADCS-ADLsev) [17], Alzheimer’s Disease Cooperative Study-Activities of Daily Living 19 Items (ADCS-ADL19) scale scores [17, 18], Alzheimer’s Disease Cooperative Study-Activities of Daily Living 23 Items (ADCS-ADL23) scale scores [18], and Bristol Activities of Daily Living Scale (BADLS) scores [19]], global function assessment [Clinician’s Interview-Based Impression of Change Plus Caregiver Input (CIBIC-Plus) [20]], stage of dementia assessment[Functional Assessment Staging instrument (FAST) [21]], rate of drug discontinuation from any cause, discontinuation because of adverse events, and discontinuation because of inefficacy. In addition, we pooled the drug side effects data.

We based our analyses on intent-to-treat (ITT) or modified ITT data (i.e., at least 1 dose or at least 1 follow-up assessment). However, we analyzed the complete set of data to ensure that as much information as possible was provided ([22]: SMMSE, NPI, and BADLS scores; [15]: SIB, ADAS-cog, MMSE, and NPI).

The meta-analysis was performed using Review Manager. To combine studies, we used the random-effects model by DerSimonian and Laird [23]. The random-effects model is more conservative than the fixed-effects model and produces a wider CI. For continuous data, we calculated Hedges’ g standardized mean difference (SMD) effect sizes and used the cut-off values for small, medium, and large effect sizes (0.2, 0.5, and 0.8, respectively) set out by Cohen [24]. If only 95% CI was reported, we converted the 95% CI to standard deviation [25]. For dichotomous data, the risk ratio (RR) was estimated along with 95% CIs. When the random-effects model showed significant differences between groups, the number needed to harm (NNH) was calculated. The NNH values were then derived from the risk difference (RD) using the formula NNH = 1/RD. We explored study heterogeneity using the *I* ^*2*^ statistic, with values of 50% or higher considered to reflect considerable heterogeneity [26]. In cases with *I* ^*2*^ values ≥50% for primary outcome measures, we conducted sensitivity analyses to determine the reasons for heterogeneity. Funnel plots were inspected visually to assess the possibility of publication bias. We also assessed the methodological qualities of the articles included in the meta-analysis on the basis of the Cochrane risk of bias criteria (Cochrane Collaboration; http://www.cochrane.org/).

## Results

### Study Characteristics

**Fig 1:**
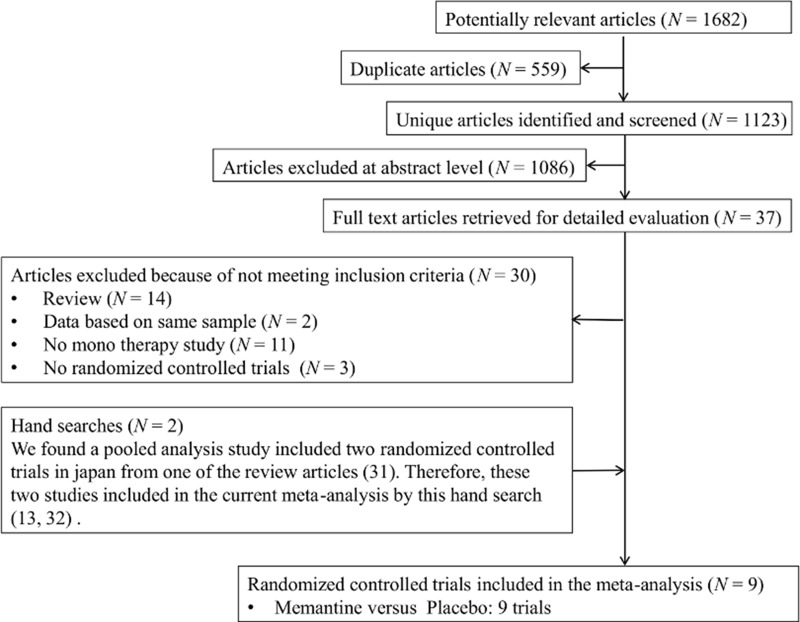
Preferred Reporting Items for Systematic reviews and Meta-Analysis (PRISMA) flow diagram.

The search yielded a total of 1647 references, of which 542 were duplicates (Fig 1). Seven RCTs testing memantine monotherapy for AD [14, 15, 22, 27–30] were included in the current meta-analysis. We excluded 1068 references after reviewing the title and abstract because these articles did not meet our criteria, and a further 30 references were excluded after full-text reviews because they were review articles (14 articles), were duplicative studies (2 articles), involved combination therapy with ChEI and memantine (11 articles), or were non-RCTs (3 articles). From a review article [31], we found a pooled analysis of 2 RCTs from Japan [13, 32]; these 2 studies were included in the current meta-analysis.

**Fig 2:**
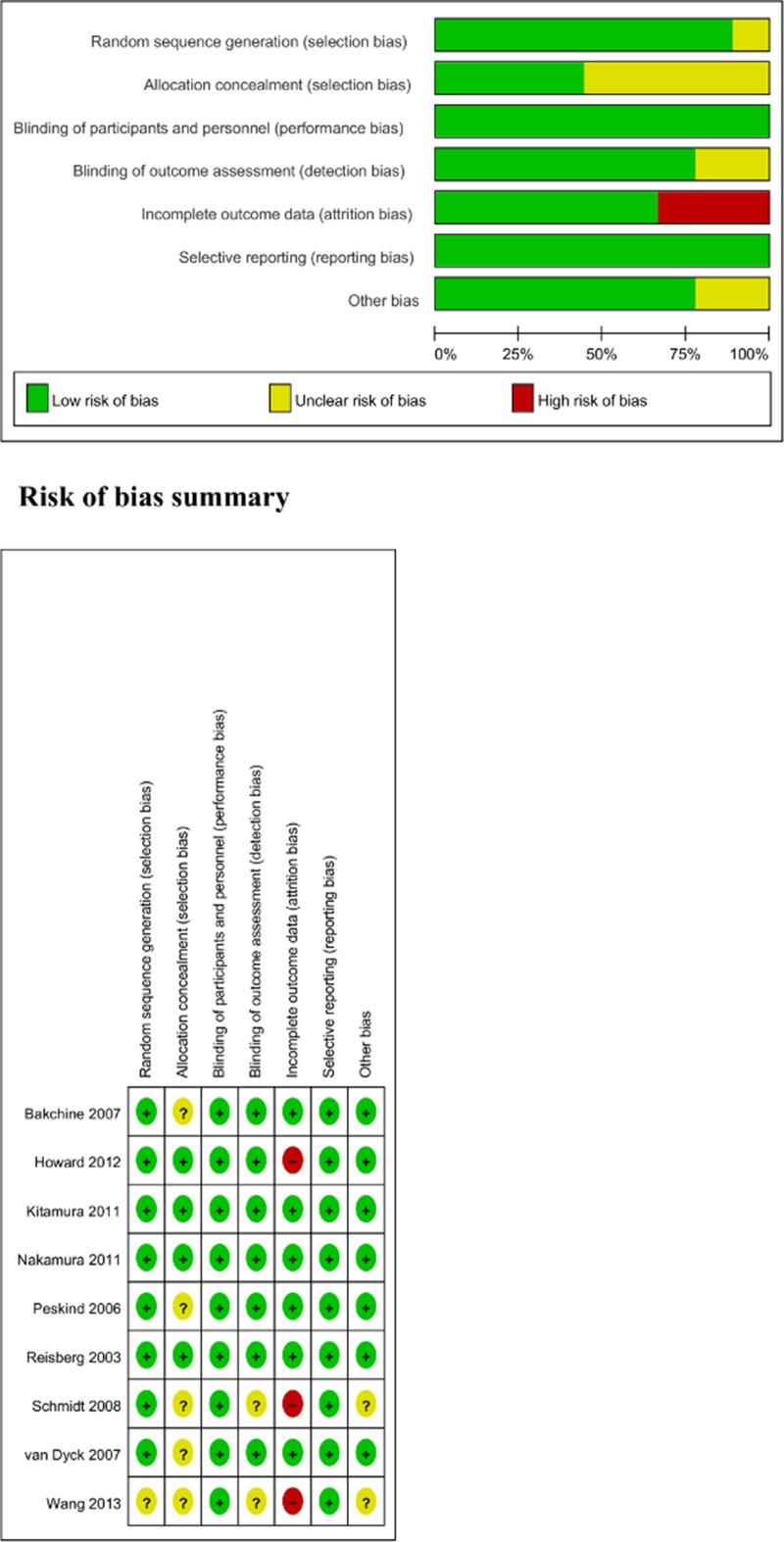
Risk of bias assessment.

In total, we identified 9 RCTs including 2433 patients with AD that met our inclusion criteria [13–15, 22, 27–30, 32]. The mean study duration was 31 weeks, with 6 trials lasting 24 weeks, 2 trials lasting 52 weeks, and 1 trial lasting 28 weeks. The total number of subjects in each study ranged from 26 to 470 patients. The mean age of the study population was 76 years. Eight out of 9 studies were sponsored by pharmaceutical companies and 2 out of 9 were published in Japanese [13, 32]. The studies were conducted in 1 or multiple countries: 3 were conducted in the United States, 2 in Japan, 1 in Austria, 1 in the United Kingdom, 1 in China, and 1 in multiple countries (Austria, Belgium, Denmark, Finland, France, Greece, Lithuania, the Netherlands, Poland, Spain, Sweden, and the United Kingdom). The characteristics of the trials included in our study are shown in Table 1. We evaluated the methodological quality of all studies using the Cochrane risk of bias criteria (Fig 2). All the studies [13–15, 22, 27–30, 32] were double blind and randomized. One study [15] did not mention the method of randomization. Five studies [14, 15, 27, 29, 30] did not mention the method of allocation concealment. Two studies [15, 30] did not mention the method of blinding outcome assessment. Finally, 3 studies [15, 22, 30] reported the complete analysis.

**Table 1:** Characteristics of included trials.

Study	Total n	Patients	Diagnosis	Duration	Age (years) (mean ± SD)	Male, %	Race (%)	Drug	n	Dose (mg/day)	Outcomes
Reisberg 2003 (USA), industry	252	AD: Outpatient (NR)	Probable AD: NINCDS-ADRDA and DSM-IV criteria	28 weeks	76.1 ± 8.07	MEM: 28, PLA: 37	MEM: White 89, Black 4, Other 7, PLA: White 91, Black 5, Other 4	MEM	126	MEM 20 mg	MEM > PLA: SIB, **ADCS-ADL (sev)**, FAST, MEM = PLA: **CIBIC-Plus**, MMSE, GDS, NPI
		Inclusion: age ≥50 years, MMSE 3–14, GDS 5–6, FAST ≥ 6a, MRI or CT consistent with a diagnosis of probable AD (within 12 months)									
		Exclusion: any neurodegenerative disorder or MDD other than AD, modified HIS <4, clinically significant coexisting medical conditions or laboratory abnormalities, receiving specific concomitant medications (anticonvulsant, antiparkinsonian, hypnotic, anxiolytic, neuroleptic, cholinomimetic, or any other investigational compounds)						PLA	126	PLA	
Peskind 2006 (USA), industry	403	AD: Outpatient (NR)	Probable AD: NINCDS-ADRDA	24 weeks	MEM: 78.0 ± 7.3, PLA: 77.0 ± 8.2	MEM: 40, PLA: 43	MEM: White 92, Other 8, PLA: White 91, Other 9	MEM	201	MEM 20 mg (fixed dose)	MEM > PLA: **ADAS-cog**, **CIBIC-Plus**, NPI, MEM = PLA: ADCS-ADL23
		Inclusion: MMSE 10–22, age ≥50 years, MRI or CT consistent with a diagnosis of probable AD (within 12 months), MADRS <22						PLA	202	PLA	
		Exclusion: significant and active pulmonary, gastrointestinal, renal, hepatic, endocrine, or cardiovascular disease; clinically significant B12 or folate deficiency; evidence of any psychiatric or neurological disorder other than probable AD; HIS <4; screening; delusions or delirium (DSM-IV); treatment with a depot neuroleptic within 6 months of screening; previous treatment with MEM; treatment within 30 days of screening with a ChEI or any investigational drug									
Study	Total n	Patients	Diagnosis	Duration	Age (years) (mean ± SD)	Male, %	Race (%)	Drug	n	Dose (mg/day)	Outcomes
van Dyck 2007 (USA), industry	350	AD: Outpatient (NR)	Probable AD: NINCDS-ADRDA	24 weeks	MEM: 78.1 ± 8.2, PLA: 78.3 ± 7.6	MEM: 27.5, PLA: 29.7	MEM: White 80, Other 20, PLA: White 82, Other 18	MEM	178	MEM 20 mg	MEM = PLA: **SIB**, **ADCS-ADL19**, CIBIC-Plus, NPI, BGP, FAST
		Inclusion: MMSE 5–14, age ≥50 years, MRI or CT consistent with a diagnosis of probable AD (within 12 months), stable dose of following concomitant medication were allowed: antihypertensives, anti-inflammatories, diuretics, laxatives, antidepressants, atypical antipsychotics, and tocopherol						PLA	172	PLA	
		Exclusion: significant and active pulmonary, gastrointestinal, renal, hepatic, endocrine, or cardiovascular disease; clinically significant B12 or folate deficiency; evidence of any psychiatric or neurological disorder other than probable AD; HIS <4; delusions or delirium (DSM-IV); active malignancy; history of substance abuse within 10 years; treatment with a depot neuroleptic within 6 months of screening; previous treatment with MEM; treatment within 30 days of screening with a ChEI or any investigational drug									
Bakchine 2007 (Austria, Belgium, Denmark, Finland, France, Greece, Lithuania, the Netherlands, Poland, Spain, Sweden, and United Kingdom), industry	470	AD: Outpatient (NR)	Probable AD: NINCDS-ADRDA and DSM-IV criteria	24 weeks	MEM: 74.0 ± 7.4, PLA: 73.3 ± 6.9	MEM: 35, PLA: 40	MEM: White 100, PLA: White 100	MEM	318	MEM 20 mg (fixed dose)	MEM = PLA: **ADAS-cog**, **CIBIC-Plus**, ADCS-ADL23, NPI
		Inclusion: MMSE 11–23, age ≥50 years, MRI or CT consistent with a diagnosis of probable AD (within 12 months); SSRIs, estrogens, anti-inflammatory drugs, β-blockers, insulin, and H2 blockers were allowed if the dose and medication had been stable for at least 3 months and were kept stable during the study; vitamin E, coenzyme Q, and atypical antipsychotics were allowed if the dose and medication had been stable for at least 30 days and kept stable during the study; atypical antipsychotics were not to be taken 3 days before a visit						PLA	152	PLA	
		Exclusion: any neurodegenerative disorder or MDD other than AD; modified HIS <4; significant coexisting medical conditions or laboratory abnormalities; receiving anticonvulsants, antiparkinsonian agents, classical and depot antipsychotics, anxiolytics, hypnotics, non-SSRI antidepressants, ChEI									
Study	Total n	Patients	Diagnosis	Duration	Age (years) (mean ± SD)	Male, %	Race (%)	Drug	n	Dose (mg/day)	Outcomes
Schmidt 2008 (Austria), industry	36	AD: Outpatient (NR)	Probable AD: NINCDS-ADRDA and DSM-IV criteria	52 weeks	MEM: 76.5 ± 4.8, PLA: 75.8 ± 5.7	MEM: 27.8, PLA: 44.4	NR	MEM	18	MEM 20 mg	NR
		Inclusion: age ≥50 years; HIS ≥4; MMSE 14–22; patients who had either failed to respond to ChEI or experienced severe side effects leading to termination of such treatment and had MMSE scores >14, which, at the time of study conduct, had excluded them from other approved antidementia treatment once ChEI had been stopped; generally good health; ChEI had to be terminated at least 4 weeks before screening; low-dose atypical neuroleptics, SSRI, non-centrally active antihypertensives, anti-inflammatory drugs, platelet antiaggregants and anticoagulants, laxatives, diuretics, and sedatives/hypnotics were permitted to continue on stable dose at least 3 months before screening						PLA	18	PLA	
		Exclusion: primary diagnosis of psychiatric disorders other than AD; cerebrovascular disease; any unstable medical condition; using anticonvulsants, antiparkinsonian agents, barbiturates, *Ginkgo biloba* and nootropics, systemic corticosteroids, and insulin									
Kitamura 2011 (Japan), industry	315	AD: Outpatient (100%)	Probable AD: NINCDS-ADRDA and DSM-IV criteria	24 weeks	MEM (20 mg): 73.2 ± 9.9, MEM (10 mg): 73.2 ± 9.6, PLA: 73.6 ± 8.9	MEM (20 mg): 26, MEM (10 mg): 35, PLA: 31	Japanese: 100%	MEM (20 mg)	100	MEM 20 mg (fixed dose)	MEM > PLA: **SIB (20 mg)**, MMSE(20 mg), FAST (20 mg), MEM = PLA: **ADCS-ADL19**, **SIB (10 mg)**, CIBIC-Plus, NPI, MMSE (10 mg), FAST (10 mg)
		Inclusion: age ≥50 years, MMSE 5–14, FAST 6a–7a						MEM (10 mg)	107	MEM 10 mg (fixed dose)	
		Exclusion: MDD (DSM-IV) other dementia with AD, other severe neurological disorder						PLA	108	PLA	
Study	Total n	Patients	Diagnosis	Duration	Age (mean ± SD)	Male, %	Race (%)	Drug	n	Dose (mg/day)	Outcomes
Nakamura 2011 (Japan), industry	432	AD: Outpatient (100%)	Probable AD: NINCDS-ADRDA and DSM-IV criteria	24 weeks	MEM: 74.4 ± 8.5, PLA: 74.9 ± 8.4	MEM: 36.2, PLA: 35.1	Japanese: 100%	MEM	221	MEM 20 mg (fixed dose)	MEM > PLA: SIB, Behave-AD, MEM = PLA: CIBIC-Plus, AST, MENFIS
		Inclusion: age ≥50 years, MMSE 5–14, FAST 6a–7a						PLA	211	PLA	
		Exclusion: MDD (DSM-IV) other dementia with AD, other severe neurological disorder									
Howard 2012 (United Kingdom), no industry	149	AD: Outpatient (NR)	Probable or possible AD: NINCDS-ADRDA criteria	52 weeks	MEM: 76.2 ± 8.9, PLA: 7.7 ± 8.0	MEM: 39, PLA: 36	MEM: White 96, Black 3, Other 1, PLA: White 97, Black 3, Other 0	MEM	76	MEM 20 mg	MEM > PLA: SMMSE, BADLS, MEM = PLA: NPI, DEMQOL-proxy, GHQ-12
		Inclusion: continuously treatment with DON for at least 3 months (treatment with DON 10 mg for at least the previous 6 weeks), SMMSE 5–13, each eligible patient’s prescribing clinician was considering a change in drug treatment on the basis of NICE guidelines at the time, discussions with the patient and caregivers, and the physician’s clinical judgment						PLA	73	PLA	
		Exclusion: severe or unstable medical conditions, current prescription of MEM, contraindications or previous adverse or allergic reactions to trial drugs									
Wang 2013 (China), industry	26	AD: Outpatient (NR)	Probable AD: NINCDS-ADRDA and DSM-IV criteria	24 weeks	MEM: 65.7 ± 12.5, PLA: 64.7 ± 11.5	MEM: 36, PLA: 36	NR	MEM	13	MEM 20 mg	MEM > PLA: SIB, MEM = PLA: MMSE, ADAS-cog, NPI
		Inclusion: age 50–90 years, MMSE 4–20, HIS ≥4, BP 160–95/95–60						PLA	13	PLA	
		Exclusion: diabetes, renal impairment, significant systemic condition, psychiatric disorder, seizures, traumatic brain injuries, using approved or investigational antidementia drugs in the previous 3 months									

AD: Alzheimer’s Disease, ADAS-cog: Alzheimer’s Disease Assessment Scale cognitive subscale, ADCS-ADL (sev): Alzheimer’s Disease Cooperative Study-Activities of Daily Living Inventory (modified for more severe dementia), Behave-AD: Behavioral Pathology in Alzheimer’s Disease Rating Scale, BGP: Behavioral Rating Scale for Geriatric Patients, BP: Blood pressure, ChEI: Cholinesterase Inhibitors, CIBIC-Plus: Clinician’s Interview-Based Impression of Change Plus Caregiver Input, CT: computed tomography, DSM-IV-TR: Diagnostic and Statistical Manual of Mental Disorders-4th edition-text revision, FAST: Functional Assessment Staging instrument, GDS: Global Deterioration Scale, GHQ-12: General Health Questionnaire 12, HIS: Hachinski Ischemic Score, MADRS: Montgomery Asberg Depression Rating Scale, MDD: Major Depressive Disorder, MEM: memantine, MENFIS: Mental Function Impairment Scale, MMSE: Mini-Mental State Examination, MRI: magnetic resonance imaging, NINCDS-ADRDA: National Institute of Neurological and Communicative Disorders and Stroke and the Alzheimer's Disease and Related Disorders Association, NPI: Neuropsychiatric Inventory, NR: Not reported, PET: positron emission tomography, PLA: placebo, SD: standard deviation, SIB: Severe Impairment Battery, SMMSE: Standardized Mini-Mental State Examination, SSRI: Selective Serotonin Reuptake Inhibitors.

### Results of the Meta-Analysis for Primary Outcomes

**Fig 3:**
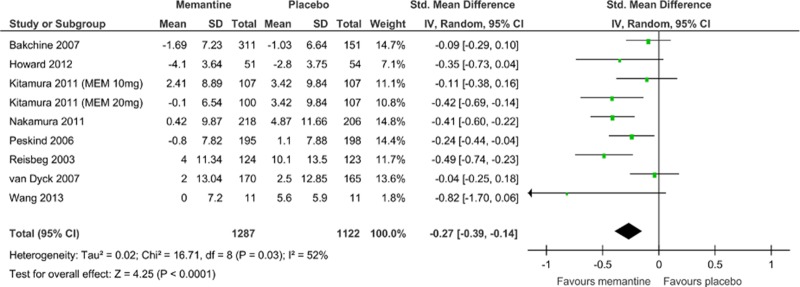
Forest plot of cognitive function (9 comparisons, n = 2409). *Negative SMD values favor memantine; positive SMD values favor placebo.†RR < 1 favors memantine; RR > 1 favors placebo.

**Fig 4:**
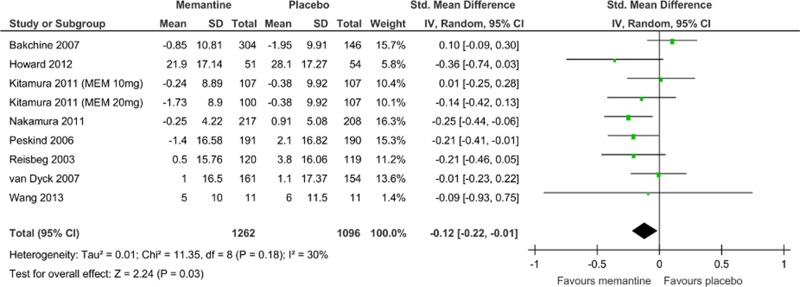
Forest plot of behavioral disturbances (9 comparisons, n = 2358). *Negative SMD values favor memantine; positive SMD values favor placebo.†RR < 1 favors memantine; RR > 1 favors placebo.

Memantine monotherapy improved cognitive function scores (SMD = −0.27, 95% CI = −0.39 to −0.14, Z = 4.25, p = 0.0001, *I* ^2^ = 52%, 9 comparisons, n = 2409; Fig 3) and behavioral disturbance scores (SMD = −0.12, 95% CI = −0.22 to −0.01, Z = 2.24, p = 0.03, *I* ^2^ = 30%, 9 comparisons, n = 2358; Fig 4). Visual inspection of the funnel plots for primary outcomes did not suggest the presence of publication bias (S2 Appendix).

### Sensitivity Analyses of Primary Outcomes

There was significant heterogeneity in cognitive function scores among the studies (*I* ^*2*^ = 52%; Fig 3). Therefore, we performed a sensitivity analysis to determine the confounding factors (Table 2), but did not detect any robust causes for the heterogeneity.

**Table 2:** Sensitivity analysis of the efficacy of memantine monotherapy (cognitive function).

Variable	Subgroup	N	n	*I* ^*2*^	SMD	95% CI	p value	Test for subgroup differences
Alzheimer’s disease stage	Mild to moderate	2	855	8	-0.17	-0.31 to -0.02	**0.02**	*I* ^*2*^ = 43.8%, p = 0.18
	Moderate to severe	7	1554	55	-0.31	-0.47 to -0.15	**0.0001**	
Neuropsychological test	ADAS-cog	2	855	8	-0.17	-0.31 to -0.02	**0.02**	*I* ^*2*^ = 0%, p = 0.39
	SMMSE	1	105	NA	-0.35	-0.73 to 0.04	0.08	
	SIB	6	1449	62	-0.31	-0.49 to -0.13	**0.0007**	
Sample size	Total n ≥ 200	7	2282	60	-0.25	-0.38 to -0.12	**0.0002**	*I* ^*2*^ = 0%, p = 0.37
	Total n < 200	2	127	0	-0.43	-0.78 to -0.07	**0.02**	
Memantine dose	Memantine 10 mg	1	214	NA	-0.11	-0.38 to 0.16	0.43	*I* ^*2*^ = 29.5%, p = 0.23
	Memantine 20 mg	8	2195	55	-0.29	-0.42 to -0.15	**0.0001**	
Method of analysis	Intention to treat	7	2282	60	-0.25	-0.38 to -0.12	**0.0002**	*I* ^*2*^ = 0%, p = 0.37
	Observed case	2	127	0	-0.43	-0.78 to -0.07	**0.02**	
Sponsorship	Industry-sponsored	1	105	NA	-0.35	-0.73 to 0.04	0.08	*I* ^*2*^ = 0%, p = 0.68
	Non-industry-sponsored	8	2304	57	-0.26	-0.40 to -0.13	**0.0001**	
Duration	≥28 weeks	8	2304	57	-0.26	-0.40 to -0.13	**0.0001**	*I* ^*2*^ = 0%, p = 0.68
	<28 weeks	1	105	NA	-0.35	-0.73 to 0.04	0.08	

ADAS-cog: Alzheimer’s Disease Assessment Scale cognitive subscale, CI: confidence interval, N: number of comparisons, n: number of patients NA: not applicable, SIB: Severe Impairment Battery, SMD: standardized mean difference, SMMSE: Standardized Mini-Mental State Examination

### Results of Meta-analysis for Secondary Outcomes

**Fig 5:**
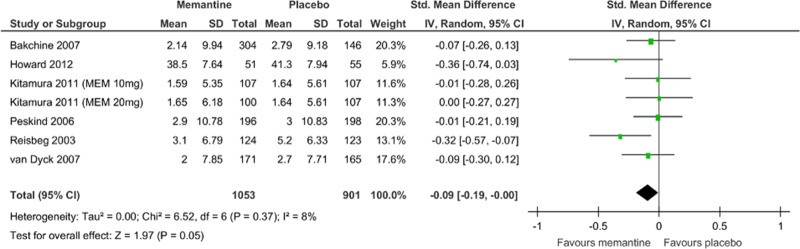
Forest plot of activity of daily living (7 comparisons, n = 1954). *Negative SMD values favor memantine; positive SMD values favor placebo.†RR < 1 favors memantine; RR > 1 favors placebo.

**Fig 6:**
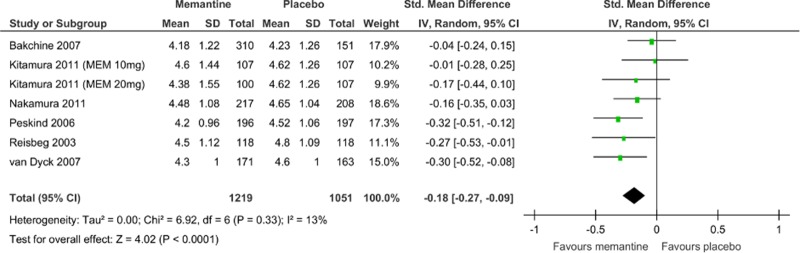
Forest plot of global function assessment (7 comparisons, n = 2270). *Negative SMD values favor memantine; positive SMD values favor placebo.†RR < 1 favors memantine; RR > 1 favors placebo.

**Fig 7:**
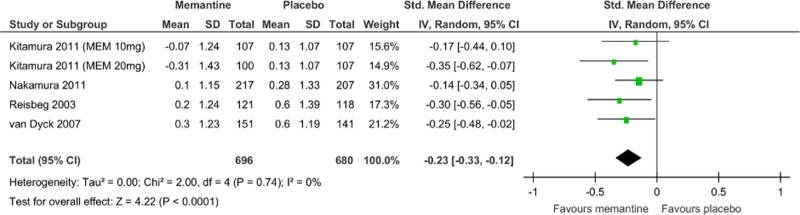
Forest plot of stage of dementia (5 comparisons, n = 1376). *Negative SMD values favor memantine; positive SMD values favor placebo.†RR < 1 favors memantine; RR > 1 favors placebo.

Memantine monotherapy significantly improved activities of daily living scores (SMD = −0.09, 95% CI = −0.19 to −0.00, Z = 1.97, p = 0.05, *I* ^2^ = 8%, 7 comparisons, n = 1954; Fig 5), global function assessment scores (SMD = −0.18, 95% CI = −0.27 to −0.09, Z = 4.02, p = 0.0001, *I* ^2^ = 13%, 7 comparisons, n = 2270; Fig 6), and stage of dementia assessment scores (SMD = −0.23, 95% CI = −0.33 to −0.12, Z = 4.22, p = 0.0001, *I* ^2^ = 0%, 5 comparisons, n = 1376; Fig 7). The data in each treatment group were simulated with no publication bias (data not shown).

**Fig 8:**
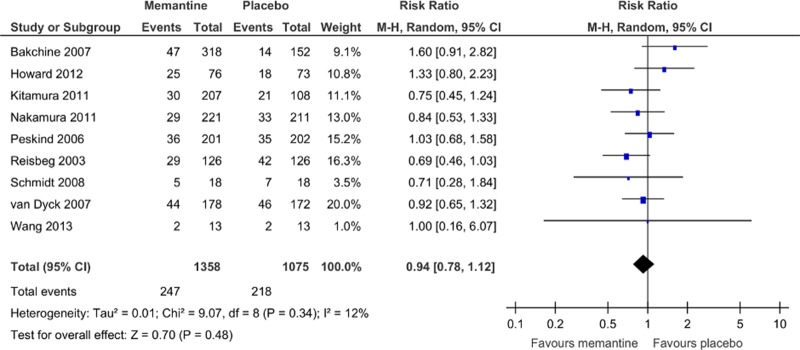
Forest plot of discontinuation due to all causes (9 studies, n = 2433). *Negative SMD values favor memantine; positive SMD values favor placebo.†RR < 1 favors memantine; RR > 1 favors placebo.

**Fig 9:**
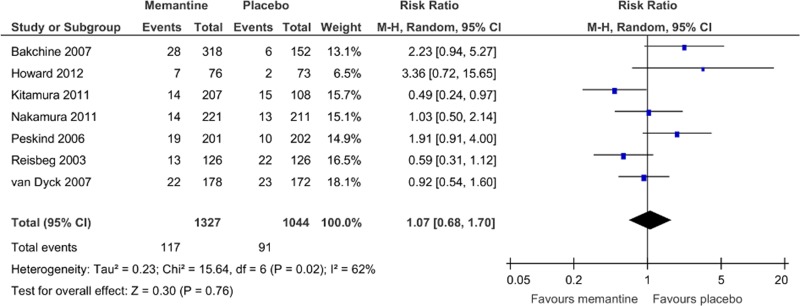
Forest plot of discontinuation due to adverse events (7 studies, n = 2371). *Negative SMD values favor memantine; positive SMD values favor placebo.†RR < 1 favors memantine; RR > 1 favors placebo.

**Fig 10:**
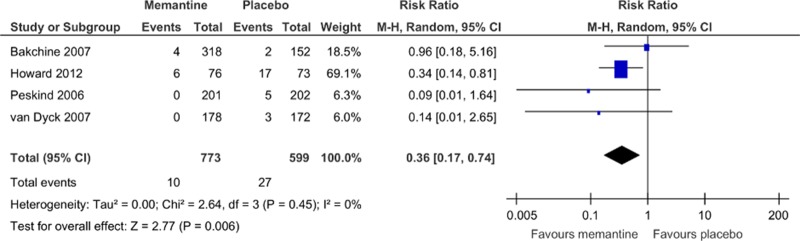
Forest plot of discontinuation due to inefficacy (4 studies, n = 1372). *Negative SMD values favor memantine; positive SMD values favor placebo.†RR < 1 favors memantine; RR > 1 favors placebo.

The rates of discontinuation because of all causes (Fig 8) and all adverse events (Fig 9) were similar between pooled memantine monotherapy and placebo groups. There was a significantly lower rate of discontinuation because of inefficacy in the pooled memantine monotherapy group (RR = 0.36, 95% CI = 0.17 to 0.74, p = 0.006, *I* ^2^ = 0%, 4 comparisons, n = 1372; NNH was not significant; Fig 10). Memantine monotherapy was also associated with a lower incidence of agitation compared with placebo (RR = 0.68, 95% CI = 0.49 to 0.94, p = 0.02, *I* ^2^ = 7%, 6 comparisons, n = 2222; NNH was not significant; S3 Appendix). No significant differences were found between groups in the incidence of all adverse events, serious adverse events, insomnia, anxiety, depression, falls, influenza-like symptoms/upper respiratory infections, dizziness, headache, urinary tract infection, peripheral edema, diarrhea, constipation, rhinitis, and death (S3 Appendix).

## Discussion

This study provides an updated, comprehensive meta-analysis of RCTs testing the efficacy of memantine monotherapy for AD. The main results indicate that memantine improves cognitive function, AD-associated behavioral disturbances, activities of daily living, global function assessment, and stage of dementia compared with placebo. However, as these effect sizes were small (SMD = −0.09 to −0.27), they suggest a limited clinical benefit. We also performed sensitivity analyses for several factors; however, we could not detect the causes of heterogeneity.

The pooled memantine treatment group exhibited a lower rate of discontinuation because of inefficacy and a lower incidence of agitation compared with the pooled placebo group. Moreover, there were no significant differences in the rates of discontinuation because of all causes, all adverse events, and individual side effects other than agitation between the memantine monotherapy and placebo groups. Therefore, memantine monotherapy does not appear to worsen the symptoms of AD and is well tolerated, exhibiting a potential beneficial role in the symptom treatment of AD.

However, these conclusions must be considered in light of several limitations. The first limitation is that we did not exclude the publication bias. The Cochrane Handbook states that tests for funnel plot asymmetry should be used only when there are at least 10 studies included in the meta-analysis, because when there are fewer studies, the power of the tests is too low to distinguish chance from real asymmetry [25]. Although the funnel plot for primary and secondary outcomes did not suggest the presence of publication bias, the number of studies included in the meta-analysis was slightly small to allow for a definitive interpretation. A second limitation is that our meta-analysis includes gray literature studies such as supported by pharmaceutical companies; we included these articles because they were published in peer-reviewed journals and represented the majority of the articles retrieved. The only non-industry-sponsored study [22] showed that memantine was marginally superior to placebo in cognitive function. Moreover, there was no significant subgroup difference between industry-sponsored and non-industry-sponsored studies (*I* ^*2*^ = 0%, p = 0.68). A third limitation is that patients with dementia are known to have poor drug compliance [33], reducing the measured effectiveness. Finally, several studies included in this meta-analysis did not report any available data on symptom scales (disease severity) and safety outcomes; therefore, the outcome results for efficacy and safety did not include data from all 9 studies.

## Conclusions

Our results suggest that memantine monotherapy is beneficial for the treatment of AD as assessed by multiple scales evaluating cognition, behavioral disturbances, activities of daily living, global function, and stage of dementia. Furthermore, memantine monotherapy appears to be well tolerated. However, the effect size in terms of efficacy outcomes was small and thus there is limited evidence of clinical benefit.

## Supporting Information

S1 PRISMA Checklist Preferred Reporting Items for Systematic Reviews and Meta-Analyses (PRISMA) checklist. (PDF)

S1 Appendix Data synthesis. (PDF)

S2 Appendix Funnel plots. (PDF)

S3 Appendix Forest plot of side effects. *Negative SMD values favor memantine; positive SMD values favor placebo. †RR < 1 favors memantine; RR > 1 favors placebo.
(PDF)
