# Supplementary material for: Memantine Monotherapy for Alzheimer’s Disease: A Systematic Review and Meta-Analysis
Source: PLoS One. 2015 Apr 10;10(4):e0123289. doi: 10.1371/journal.pone.0123289 (PMC4393306; doi:10.1371/journal.pone.0123289)
Supplement: S1 Appendix — (PDF) [file pone.0123289.s002.pdf]

Supplementary appendix 1. Date synthesis.

Supplementary table 1 Data synthesis

		Reisberg 2003	Peskind 2006	van Dyck 2007	Bakchine 2007	Schmidt 2008	Kitamura 2011	Nakamura 2011	Howard 2012	Wang 2013
Memantine VS Placebo	Cognitive function	change scores of SIB	change scores of ADAS-cog	change scores of SIB	change scores of ADAS-cog		change scores of SIB	change scores of SIB	endpoint scores of SMMSE	change scores of SIB
	Behavioral disturbances	change scores of NPI	change scores of NPI	change scores of NPI	change scores of NPI		change scores of NPI	change scores of Behave-AD	endpoint scores of NPI	change scores of NPI
	Activity of daily living	change scores of ADCS-ADLsev	change scores of ADCS-ADL23	change scores of ADCS-ADL19	change scores of ADCS-ADL23		change scores of ADCS-ADL19		endpoint scores of BADLS	
	Global assessment	change scores of CIBIC-plus	change scores of CIBIC-plus	change scores of CIBIC-plus	change scores of CIBIC-plus		change scores of CIBIC-plus	change scores of CIBIC-plus		
	Stage of dementia	change scores of FAST		change scores of FAST			change scores of FAST	change scores of FAST		

ADAS-cog: Alzheimer's Disease Assessment Scale cognitive subscale, ADCS-ADL (sev): Alzheimer’s Disease Cooperative Study–Activities of Daily Living (modified for more severe dementia), Behave-AD: Behavioral Psychology in Alzheimer's Disease Rating Scale, CIBIC-Plus: Clinician’s Interview-Based Impression of Change Plus Caregiver Input, FAST: Functional Assessment Staging instrument, NPI: neuro-psychiatric inventory, SIB: Severe Impairment Battery, SMMSE: Standardized Mini–Mental State Examination
