# Supplementary figures and images for: Memantine Monotherapy for Alzheimer’s Disease: A Systematic Review and Meta-Analysis
Source: PLoS One. 2015 Apr 10;10(4):e0123289. doi: 10.1371/journal.pone.0123289 (PMC4393306; doi:10.1371/journal.pone.0123289)

## Supplementary appendix 2. Funnel plots

### Cognitive function

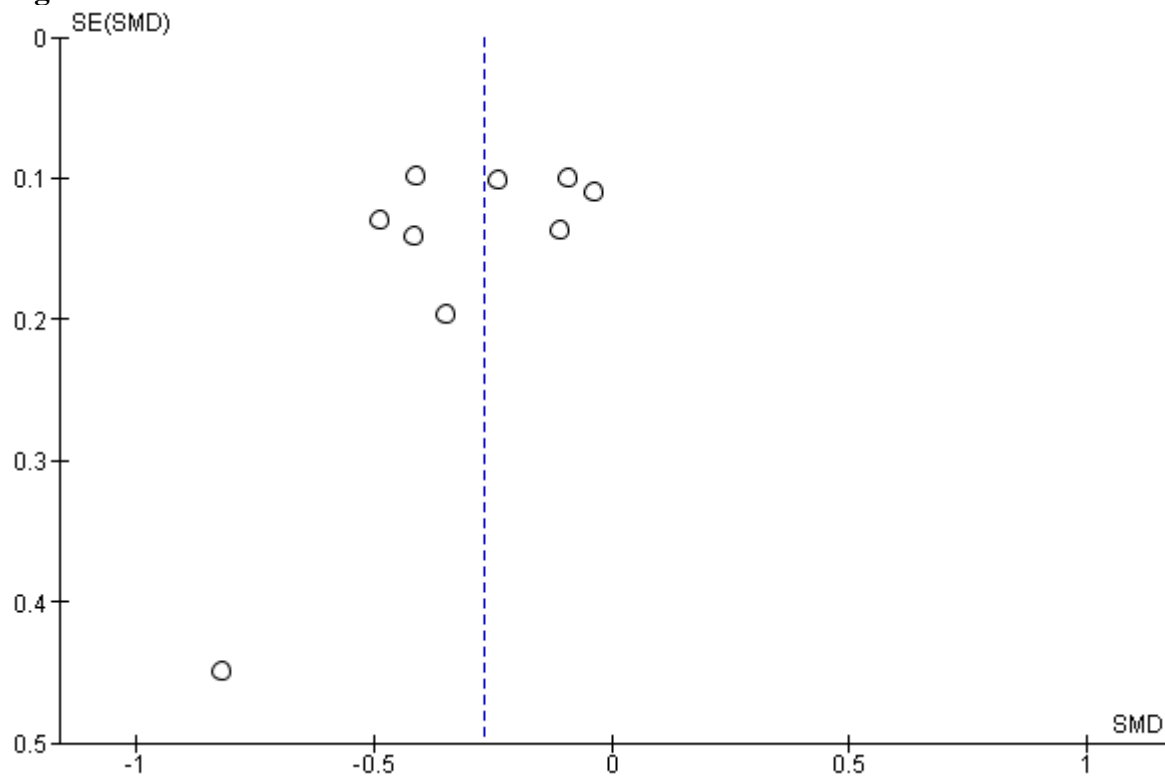

### Behavioral disturbances

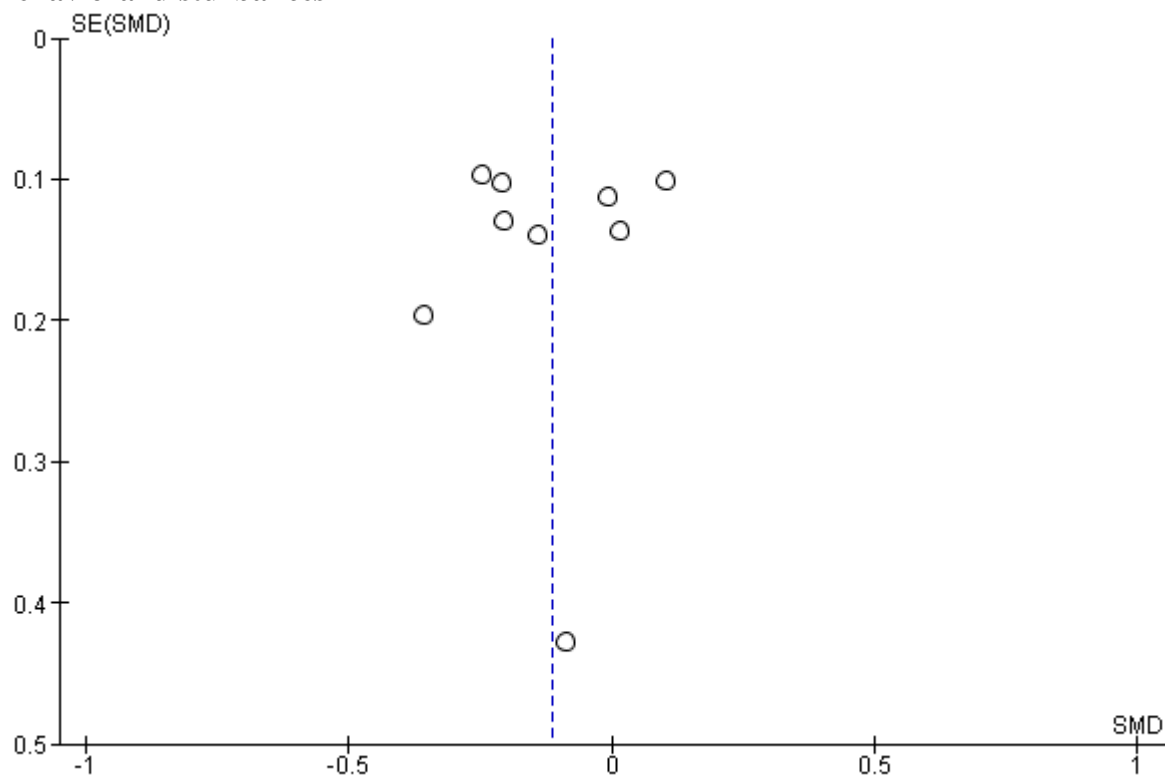

Supplement: S2 Appendix — (PDF) [file pone.0123289.s003.pdf]
