# Supplementary material for: Memantine Monotherapy for Alzheimer’s Disease: A Systematic Review and Meta-Analysis
Source: PLoS One. 2015 Apr 10;10(4):e0123289. doi: 10.1371/journal.pone.0123289 (PMC4393306; doi:10.1371/journal.pone.0123289)

Supplementary appendix 3. Forest plot of side effects.

All adverse events (6 studies, n = 2222)

	Memantine		Placebo			Risk Ratio
Study or Subgroup	Events	Total	Events	Total	Weight	M-H, Random, 95% CI
Bakchine 2007	178	318	80	152	7.4%	1.06 [0.89, 1.27]
Kitamura 2011	155	207	78	108	11.9%	1.04 [0.90, 1.19]
Nakamura 2011	180	221	167	211	27.1%	1.03 [0.94, 1.13]
Peskind 2006	149	201	143	202	16.3%	1.05 [0.93, 1.18]
Reisbeg 2003	106	126	109	126	22.6%	0.97 [0.88, 1.08]
van Dyck 2007	131	178	125	172	14.7%	1.01 [0.89, 1.15]
**Total (95% CI)**		**1251**		**971**	**100.0%**	**1.02 [0.97, 1.07]**
Total events	899		702			

Heterogeneity: Tau² = 0.00; Chi² = 1.41, df = 5 (P = 0.92); I² = 0%
Test for overall effect: Z = 0.79 (P = 0.43)

Risk Ratio
M-H, Random, 95% CI

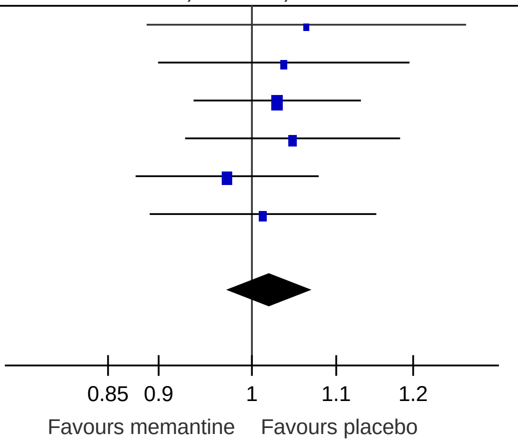

Serious adverse events (7 studies, n = 2371)

	Memantine		Placebo			Risk Ratio
Study or Subgroup	Events	Total	Events	Total	Weight	M-H, Random, 95% CI
Bakchine 2007	33	318	9	152	6.4%	1.75 [0.86, 3.57]
Howard 2012	40	76	46	73	42.7%	0.84 [0.63, 1.10]
Kitamura 2011	17	207	11	108	6.3%	0.81 [0.39, 1.66]
Nakamura 2011	26	221	24	211	12.0%	1.03 [0.61, 1.74]
Peskind 2006	20	201	20	202	9.4%	1.00 [0.56, 1.81]
Reisbeg 2003	16	126	23	126	9.4%	0.70 [0.39, 1.25]
van Dyck 2007	26	178	29	172	13.8%	0.87 [0.53, 1.41]
**Total (95% CI)**		**1327**		**1044**	**100.0%**	**0.90 [0.75, 1.08]**
Total events	178		162			

Heterogeneity: Tau² = 0.00; Chi² = 5.05, df = 6 (P = 0.54); I² = 0%
Test for overall effect: Z = 1.12 (P = 0.26)

Risk Ratio
M-H, Random, 95% CI

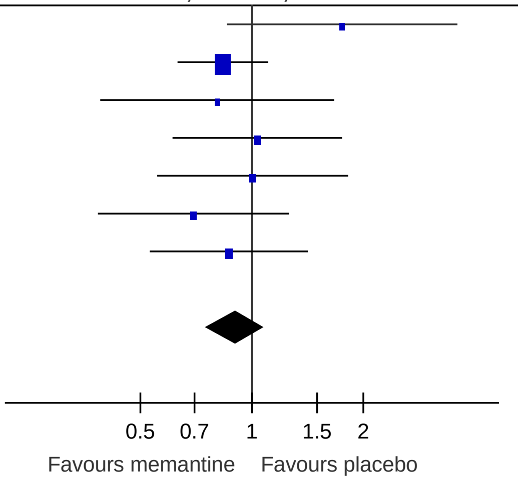

Agitation (6 studies, n = 2222)

	Memantine		Placebo			Risk Ratio
Study or Subgroup	Events	Total	Events	Total	Weight	M-H, Random, 95% CI
Bakchine 2007	5	318	7	152	7.9%	0.34 [0.11, 1.06]
Kitamura 2011	3	207	2	108	3.3%	0.78 [0.13, 4.61]
Nakamura 2011	3	221	2	211	3.3%	1.43 [0.24, 8.49]
Peskind 2006	15	201	12	202	17.9%	1.26 [0.60, 2.62]
Reisbeg 2003	23	126	40	126	41.7%	0.57 [0.37, 0.90]
van Dyck 2007	16	178	24	172	25.9%	0.64 [0.35, 1.17]
**Total (95% CI)**		**1251**		**971**	**100.0%**	**0.68 [0.49, 0.94]**
Total events	65		87			

Heterogeneity: Tau² = 0.01; Chi² = 5.39, df = 5 (P = 0.37); I² = 7%
Test for overall effect: Z = 2.33 (P = 0.02)

Risk Ratio
M-H, Random, 95% CI

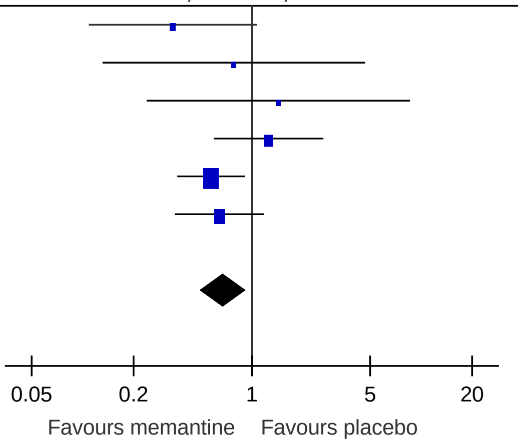

Insomnia (4 studies, n = 1349)

	Memantine		Placebo			Risk Ratio
**Study or Subgroup**	**Events**	**Total**	**Events**	**Total**	**Weight**	**M-H, Random, 95% CI**
Kitamura 2011	9	207	5	108	17.7%	0.94 [0.32, 2.73]
Nakamura 2011	14	221	11	211	34.4%	1.22 [0.56, 2.62]
Reisbeg 2003	13	126	10	126	32.7%	1.30 [0.59, 2.85]
van Dyck 2007	4	178	9	172	15.1%	0.43 [0.13, 1.37]
**Total (95% CI)**		**732**		**617**	**100.0%**	**1.01 [0.65, 1.59]**
Total events	40		35			

Heterogeneity: Tau² = 0.00; Chi² = 2.74, df = 3 (P = 0.43); I² = 0%

Test for overall effect: Z = 0.06 (P = 0.95)

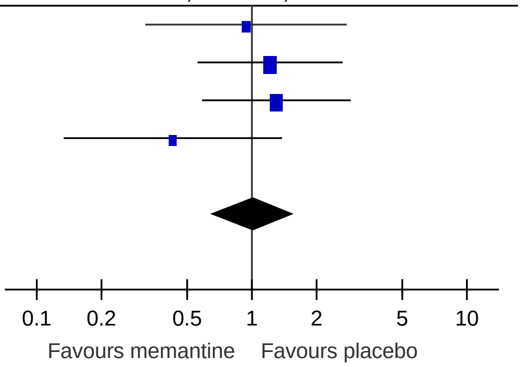

Anxiety (3 studies, n = 1097)

	Memantine		Placebo			Risk Ratio
**Study or Subgroup**	**Events**	**Total**	**Events**	**Total**	**Weight**	**M-H, Random, 95% CI**
Kitamura 2011	3	207	4	108	35.1%	0.39 [0.09, 1.72]
Nakamura 2011	0	221	1	211	10.4%	0.32 [0.01, 7.77]
van Dyck 2007	10	178	6	172	54.6%	1.61 [0.60, 4.34]
**Total (95% CI)**		**606**		**491**	**100.0%**	**0.83 [0.28, 2.46]**
Total events	13		11			

Heterogeneity: Tau² = 0.31; Chi² = 2.92, df = 2 (P = 0.23); I² = 32%

Test for overall effect: Z = 0.34 (P = 0.74)

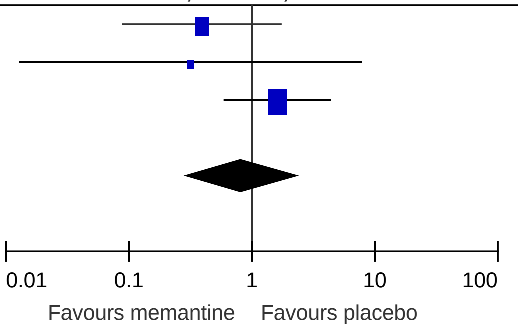

Depression (3 studies, n = 1068)

	Memantine		Placebo			Risk Ratio
**Study or Subgroup**	**Events**	**Total**	**Events**	**Total**	**Weight**	**M-H, Random, 95% CI**
Kitamura 2011	1	207	0	108	10.6%	1.57 [0.06, 38.27]
Peskind 2006	4	201	10	202	43.5%	0.40 [0.13, 1.26]
van Dyck 2007	9	178	5	172	46.0%	1.74 [0.59, 5.09]
**Total (95% CI)**		**586**		**482**	**100.0%**	**0.91 [0.30, 2.78]**
Total events	14		15			

Heterogeneity: Tau² = 0.40; Chi² = 3.48, df = 2 (P = 0.18); I² = 43%

Test for overall effect: Z = 0.16 (P = 0.87)

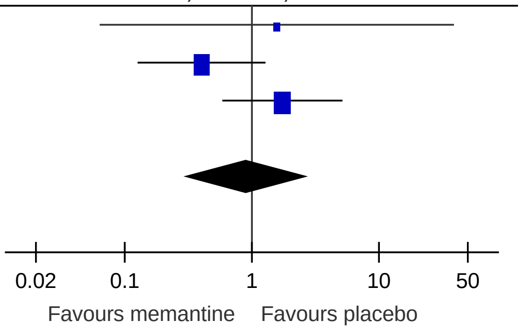

Fall (5 studies, n = 1649)

Study or Subgroup	Memantine Events	Memantine Total	Placebo Events	Placebo Total	Weight	Risk Ratio M-H, Random, 95% CI
Howard 2012	8	76	12	73	14.0%	0.64 [0.28, 1.48]
Kitamura 2011	11	207	8	108	12.5%	0.72 [0.30, 1.73]
Nakamura 2011	25	221	25	211	35.8%	0.95 [0.57, 1.61]
Peskind 2006	15	201	15	202	20.5%	1.00 [0.50, 2.00]
van Dyck 2007	10	178	17	172	17.2%	0.57 [0.27, 1.21]
**Total (95% CI)**		**883**		**766**	**100.0%**	**0.81 [0.59, 1.10]**
Total events	69		77			

Heterogeneity: Tau² = 0.00; Chi² = 1.99, df = 4 (P = 0.74); I² = 0%

Test for overall effect: Z = 1.36 (P = 0.17)

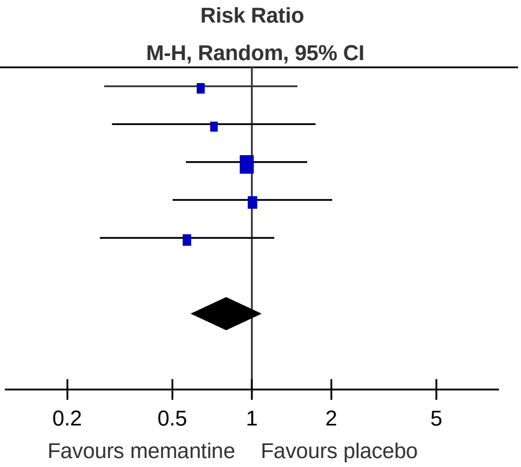

Influenza-like symptoms/Upper respiratory infection (5 studies, n = 1649)

Study or Subgroup	Memantine Events	Memantine Total	Placebo Events	Placebo Total	Weight	Risk Ratio M-H, Random, 95% CI
Howard 2012	7	76	6	73	21.0%	1.12 [0.40, 3.18]
Kitamura 2011	10	207	5	108	20.8%	1.04 [0.37, 2.98]
Nakamura 2011	6	221	3	211	13.0%	1.91 [0.48, 7.54]
Peskind 2006	4	201	12	202	18.8%	0.33 [0.11, 1.02]
van Dyck 2007	10	178	8	172	26.4%	1.21 [0.49, 2.99]
**Total (95% CI)**		**883**		**766**	**100.0%**	**0.96 [0.57, 1.63]**
Total events	37		34			

Heterogeneity: Tau² = 0.06; Chi² = 4.77, df = 4 (P = 0.31); I² = 16%

Test for overall effect: Z = 0.14 (P = 0.89)

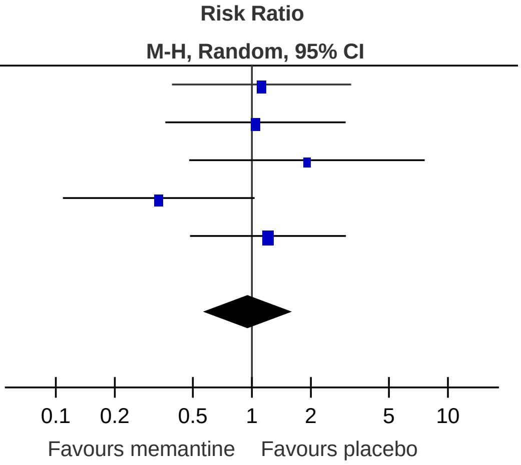

Dizziness (5 studies, n = 1970)

Study or Subgroup	Memantine Events	Memantine Total	Placebo Events	Placebo Total	Weight	Risk Ratio M-H, Random, 95% CI
Bakchine 2007	17	318	6	152	24.7%	1.35 [0.54, 3.37]
Kitamura 2011	6	207	1	108	4.6%	3.13 [0.38, 25.67]
Nakamura 2011	7	221	3	211	11.4%	2.23 [0.58, 8.50]
Peskind 2006	10	201	9	202	26.5%	1.12 [0.46, 2.69]
van Dyck 2007	12	178	11	172	32.7%	1.05 [0.48, 2.32]
**Total (95% CI)**		**1125**		**845**	**100.0%**	**1.30 [0.83, 2.05]**
Total events	52		30			

Heterogeneity: Tau² = 0.00; Chi² = 1.70, df = 4 (P = 0.79); I² = 0%

Test for overall effect: Z = 1.15 (P = 0.25)

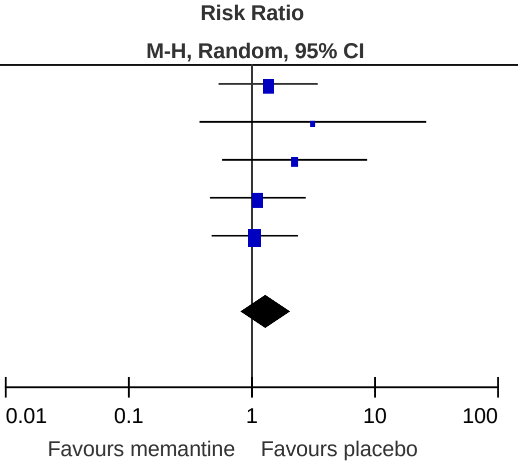

Headache (5 studies, n = 1970)

Study or Subgroup	Memantine Events	Memantine Total	Placebo Events	Placebo Total	Weight	Risk Ratio M-H, Random, 95% CI
Bakchine 2007	18	318	3	152	19.9%	2.87 [0.86, 9.59]
Kitamura 2011	5	207	2	108	14.0%	1.30 [0.26, 6.61]
Nakamura 2011	5	221	5	211	19.6%	0.95 [0.28, 3.25]
Peskind 2006	13	201	9	202	27.5%	1.45 [0.63, 3.32]
van Dyck 2007	3	178	11	172	19.0%	0.26 [0.07, 0.93]
**Total (95% CI)**		**1125**		**845**	**100.0%**	**1.09 [0.52, 2.30]**
Total events	44		30			

Heterogeneity: Tau² = 0.35; Chi² = 7.88, df = 4 (P = 0.10); I² = 49%

Test for overall effect: Z = 0.23 (P = 0.82)

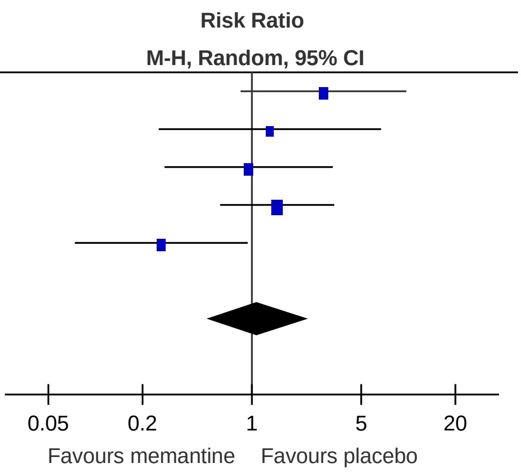

Urinary tract infection (5 studies, n = 1498)

Study or Subgroup	Memantine Events	Memantine Total	Placebo Events	Placebo Total	Weight	Risk Ratio M-H, Random, 95% CI
Howard 2012	8	76	2	73	14.7%	3.84 [0.84, 17.49]
Kitamura 2011	5	207	4	108	18.1%	0.65 [0.18, 2.38]
Nakamura 2011	2	221	4	211	12.6%	0.48 [0.09, 2.58]
Reisbeg 2003	7	126	17	126	28.1%	0.41 [0.18, 0.96]
van Dyck 2007	9	178	9	172	26.6%	0.97 [0.39, 2.38]
**Total (95% CI)**		**808**		**690**	**100.0%**	**0.79 [0.39, 1.60]**
Total events	31		36			

Heterogeneity: Tau² = 0.27; Chi² = 7.05, df = 4 (P = 0.13); I² = 43%

Test for overall effect: Z = 0.65 (P = 0.52)

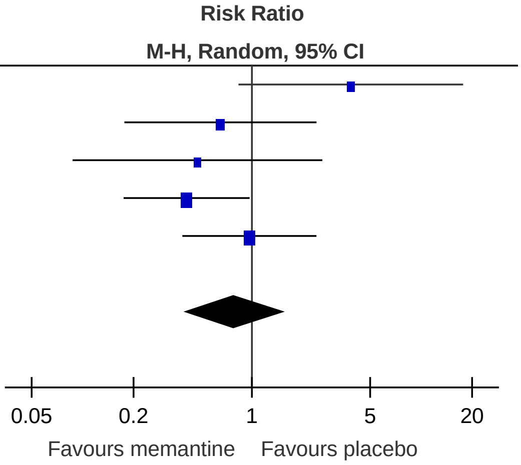

Peripheral edema (3 studies, n = 1097)

Study or Subgroup	Memantine Events	Memantine Total	Placebo Events	Placebo Total	Weight	Risk Ratio M-H, Random, 95% CI
Kitamura 2011	14	207	7	108	40.0%	1.04 [0.43, 2.51]
Nakamura 2011	3	221	7	211	19.5%	0.41 [0.11, 1.56]
van Dyck 2007	12	178	8	172	40.5%	1.45 [0.61, 3.46]
**Total (95% CI)**		**606**		**491**	**100.0%**	**0.99 [0.53, 1.86]**
Total events	29		22			

Heterogeneity: Tau² = 0.05; Chi² = 2.42, df = 2 (P = 0.30); I² = 17%

Test for overall effect: Z = 0.02 (P = 0.98)

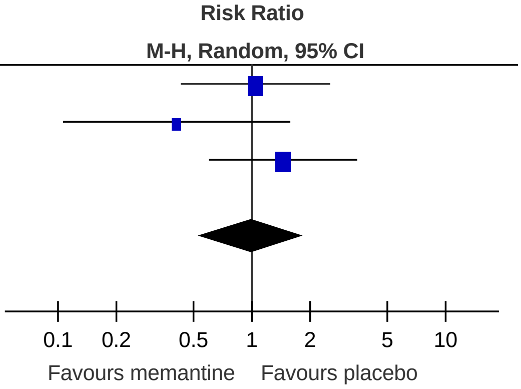

Diarrhea (4 studies, n = 1349)

Study or Subgroup	Memantine Events	Memantine Total	Placebo Events	Placebo Total	Weight	Risk Ratio M-H, Random, 95% CI
Kitamura 2011	7	207	4	108	13.7%	0.91 [0.27, 3.05]
Nakamura 2011	10	221	13	211	31.0%	0.73 [0.33, 1.64]
Reisbeg 2003	12	126	10	126	31.0%	1.20 [0.54, 2.68]
van Dyck 2007	10	178	8	172	24.3%	1.21 [0.49, 2.99]
**Total (95% CI)**		**732**		**617**	**100.0%**	**0.99 [0.64, 1.55]**
Total events	39		35			

Heterogeneity: Tau² = 0.00; Chi² = 0.96, df = 3 (P = 0.81); I² = 0%

Test for overall effect: Z = 0.02 (P = 0.98)

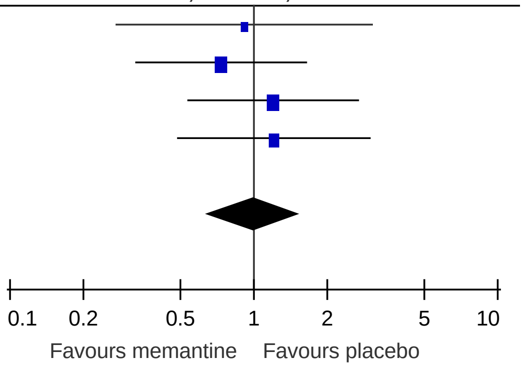

Constipation (3 studies, n = 1097)

Study or Subgroup	Memantine Events	Memantine Total	Placebo Events	Placebo Total	Weight	Risk Ratio M-H, Random, 95% CI
Kitamura 2011	19	207	10	108	26.8%	0.99 [0.48, 2.06]
Nakamura 2011	30	221	23	211	55.0%	1.25 [0.75, 2.07]
van Dyck 2007	11	178	8	172	18.2%	1.33 [0.55, 3.22]
**Total (95% CI)**		**606**		**491**	**100.0%**	**1.19 [0.81, 1.73]**
Total events	60		41			

Heterogeneity: Tau² = 0.00; Chi² = 0.33, df = 2 (P = 0.85); I² = 0%

Test for overall effect: Z = 0.88 (P = 0.38)

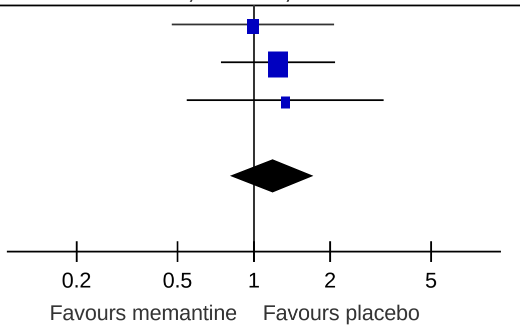

Rhinitis (3 studies, n = 1217)

Study or Subgroup	Memantine Events	Memantine Total	Placebo Events	Placebo Total	Weight	Risk Ratio M-H, Random, 95% CI
Bakchine 2007	14	318	7	152	20.2%	0.96 [0.39, 2.32]
Kitamura 2011	34	207	12	108	32.7%	1.48 [0.80, 2.74]
Nakamura 2011	32	221	42	211	47.1%	0.73 [0.48, 1.11]
**Total (95% CI)**		**746**		**471**	**100.0%**	**0.97 [0.61, 1.54]**
Total events	80		61			

Heterogeneity: Tau² = 0.07; Chi² = 3.50, df = 2 (P = 0.17); I² = 43%

Test for overall effect: Z = 0.13 (P = 0.89)

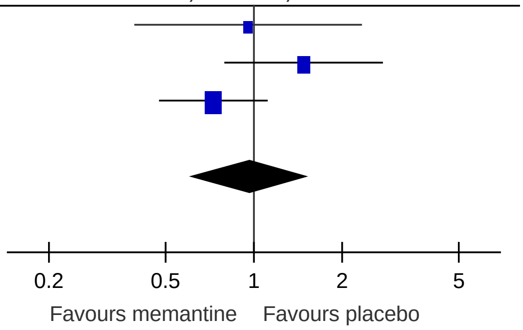

Death (7 studies, n = 2371)

	Memantine		Placebo			Risk Ratio
Study or Subgroup	Events	Total	Events	Total	Weight	M-H, Random, 95% CI
Bakchine 2007	5	318	2	152	12.0%	1.19 [0.23, 6.09]
Howard 2012	10	76	9	73	45.0%	1.07 [0.46, 2.48]
Kitamura 2011	1	207	1	108	4.2%	0.52 [0.03, 8.26]
Nakamura 2011	5	221	2	211	12.0%	2.39 [0.47, 12.17]
Peskind 2006	1	201	1	202	4.2%	1.00 [0.06, 15.96]
Reisbeg 2003	1	126	4	126	6.7%	0.25 [0.03, 2.21]
van Dyck 2007	5	178	3	172	15.9%	1.61 [0.39, 6.64]
**Total (95% CI)**		**1327**		**1044**	**100.0%**	**1.12 [0.64, 1.96]**
Total events	28		22			

Heterogeneity: Tau² = 0.00; Chi² = 3.23, df = 6 (P = 0.78); I² = 0%

Test for overall effect: Z = 0.38 (P = 0.70)

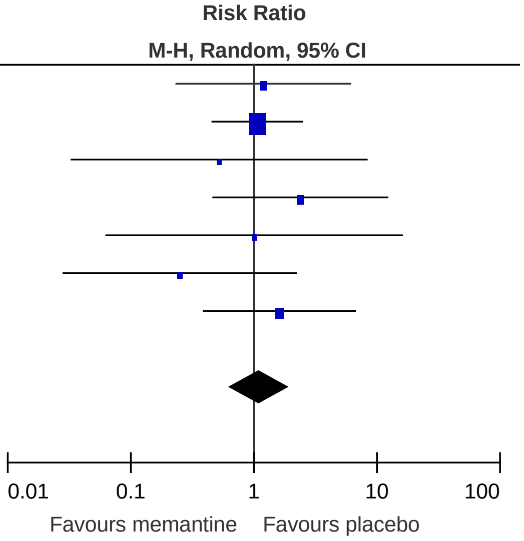

Supplement: S3 Appendix — *Negative SMD values favor memantine; positive SMD values favor placebo. †RR < 1 favors memantine; RR > 1 favors placebo. (PDF) [file pone.0123289.s004.pdf]
